# *PSEN1* variants in Korean patients with clinically suspicious early-onset familial Alzheimer’s disease

**DOI:** 10.1038/s41598-020-59829-z

**Published:** 2020-02-26

**Authors:** Young-Eun Kim, Hanna Cho, Hee Jin Kim, Duk L. Na, Sang Won Seo, Chang-Seok Ki

**Affiliations:** 1 Department of Laboratory Medicine, Hanyang University College of Medicine, Seoul, Korea; 2 Department of Neurology, Gangnam Severance Hospital, Yonsei University College of Medicine, Seoul, Korea; 3 Department of Neurology, Hanyang University College of Medicine, Seoul, Korea; 4 Department of Neurology, Samsung Medical Center, Sungkyunkwan University School of Medicine, Seoul, Korea; 5 GC Genome, Yongin, Korea

**Keywords:** Molecular medicine, Neurology

## Abstract

Pathogenic variants in the *PSEN1* gene are known to be the most common cause of early-onset Alzheimer’s disease but there are few data on the frequency and spectrum of *PSEN1* variants in Korea. In this study, we investigated *PSEN1* variants in a consecutive series of clinically suspicious early-onset familial AD (EOFAD) Korean patients and their clinical characteristics and imaging findings. From January 2007 to December 2013, EOFAD patients with very early onset AD (<50 yr), early onset AD (<60 yr) with two or more relatives with AD, and early onset AD (<60 yr) with one or more first-degree relatives with very early onset AD (<50 yr) were enrolled in this study. Sequence analysis of the *PSEN1* gene was performed by Sanger sequencing. Neuroimaging data and conventional brain MRIs and FDG-PET and/or [^11^C] PiB-PET scans were analyzed in patients with *PSEN1* variants. Among the 28 patients with EOFAD, six (21.4%, 6/28) patients had pathogenic or likely pathogenic variants in the *PSEN1* gene. Two pathogenic variants were p.Glu120Lys and p.Ser170Phe and four likely pathogenic variants were p.Thr119Ile, p.Tyr159Cys, p.Leu282Pro, and p.Ala285Ser. Two patients had variants of unknown significance, p.Tyr389His and p.Tyr389Ser. EOFAD patients with *PSEN1* variants showed early AD onset, frequent visuospatial dysfunction, movement disorders, and rapid disease progression. Brain MRIs revealed diffuse cortical atrophy, including parietal lobe atrophy, and/or hippocampal atrophy. FDG-PET scans also revealed significant hypometabolism in the bilateral temporo-parietal regions. Our findings provide insight to better understand the genetic background of Korean EOFAD patients.

## Introduction

Alzheimer’s disease (AD, OMIM# 104300) is a progressive neurodegenerative disease characterized by memory loss and personality changes. AD is the most common type of dementia in the elderly, representing 50 to 70% of dementia cases^1^. AD can be classified into two subtypes, early-onset AD and late-onset AD, based on the age at onset^2^. A family history of AD is more frequent in early-onset AD (7%) than in late-onset AD (1.7%)^3–5^.

*PSEN1* mutations are essential components of the γ-secretase complex and mutations in *PSEN1* impair the γ-secretase cleavage system of amyloid precursor protein (APP) into beta amyloid (Aβ) fragments, resulting in an increased ratio of Aβ_42_ to Aβ_40_^6^. Consequently, it can form insoluble toxic fibrils and lead to the aggregation of toxic Aβ deposited in extracellular amyloid plaques^6,7^.

It is important to characterize the preclinical stages of AD in the model of autosomal dominant Alzheimer’s disease with the expectation that future treatment may target the specific disease protein. The Dominantly Inherited Alzheimer Network (DIAN) was established for the purpose of identifying individuals at risk for autosomal dominant Alzheimer’s disease^8^. Its primary aim is to investigate the temporal ordering of AD pathophysiological changes which occur in asymptomatic and symptomatic carriers and non-carriers of autosomal dominant Alzheimer’s disease mutations in genes such as *PSEN1*, *PSEN2*, and *APP*.

Therefore, in this study, we investigated the frequency and spectrum of *PSEN1* variants in a consecutive series of clinically suspicious early-onset familial AD (EOFAD) Korean patients to determine the *PSEN1* genetic background of EOFAD.

## Methods

### Participants

Among 1,101 patients with AD who visited the Memory Disorder Clinic at Samsung Medical Center from January 2007 to December 2013, 149 patients were classified into the early-onset AD (EOAD) group (onset age < 60 years). Among them, we selected 28 clinically suspicious early-onset familial AD (EOFAD) patients using the following criteria: 1) very early disease onset (<50 years old), 2) early disease onset (<60 years old) with two or more affected relatives, and 3) early disease onset (<60 years old) with one or more affected first-degree relatives with early onset dementia (<60 years old). Age at onset was defined as the age at which the earliest episode of cognitive decline was reported.

All patients met the criteria for probable Alzheimer’s disease proposed by the National Institute of Neurological and Communicative Disorders and Stroke and the Alzheimer’s Disease and Related Disorders Association (NINCDS-ADRDA)^9^ and also met the diagnosis of dementia due to Alzheimer’s disease^10^. At the initial visit, the patients were evaluated by a neurologist and underwent clinical interviews, a neurological examination, a battery of neuropsychological tests called the Seoul Neuropsychological Screening Battery (SNSB)^11^, and conventional brain MRI scans including T1 and T2 weighted imaging. Potential secondary causes of cognitive deficits were ruled out by laboratory tests, including complete blood counts, blood chemistry, vitamin B12 levels, folate levels, syphilis serology, and thyroid function tests. This study was approved by the Institutional Review Board of Samsung Medical Center. Informed consent was obtained from all subjects. All research was performed in accordance with relevant guidelines.

### Genetic analysis

Genomic DNA (gDNA) was extracted from peripheral blood leukocytes using Wizard Genomic DNA Purification kits according to the manufacturer’s instructions (Promega, Madison, WI, USA). All exons and exon-intron boundaries of the *PSEN1* gene were amplified by PCR using primers designed by the authors (available on request). PCR was performed with a thermal cycler model GeneAmp PCR system 9700 (Applied Biosystems, Foster City, CA, USA). Sanger sequencing was performed with Big Dye Terminator Cycle Sequencing Ready Reaction kits (Applied Biosystems) on an ABI 3130*xl* Genetic Analyzer (Applied Biosystems). *PSEN1* cDNA nucleotides were numbered according to the reference sequence GenBank accession number NM_000021.4. *APOE* genotyping was performed using TaqMan SNP Genotyping Assays (Applied Biosystems, Forster City, CA, USA) on a 7500 Fast Real-Time PCR System (Applied Biosystems) according to the manufacturer’s instructions. All variants were classified according to pathogenic criteria of the American College of Medical Genetics and Genomics and the Association for Molecular Pathology (ACMG-AMP) guidelines^12^. In addition, we compared the results of *PSEN1* genetic analyses to 100 ethnically-matched normal control subjects from the Korean Genome Analysis Project (4845–301), the Korean Genome and Epidemiology Study (4851–302), and the Korean Biobank Project (4851–307, KBP-2014-031) supported by the Korean Center for Disease Control and Prevention, Republic of Korea. Four hundred exome data were also used as in-house controls. Additional analysis of *PSEN2* and *APP* genes in the patients could not be performed because the samples were unavailable.

## Results

### Identification of PSEN1 variants

**Figure 1:**
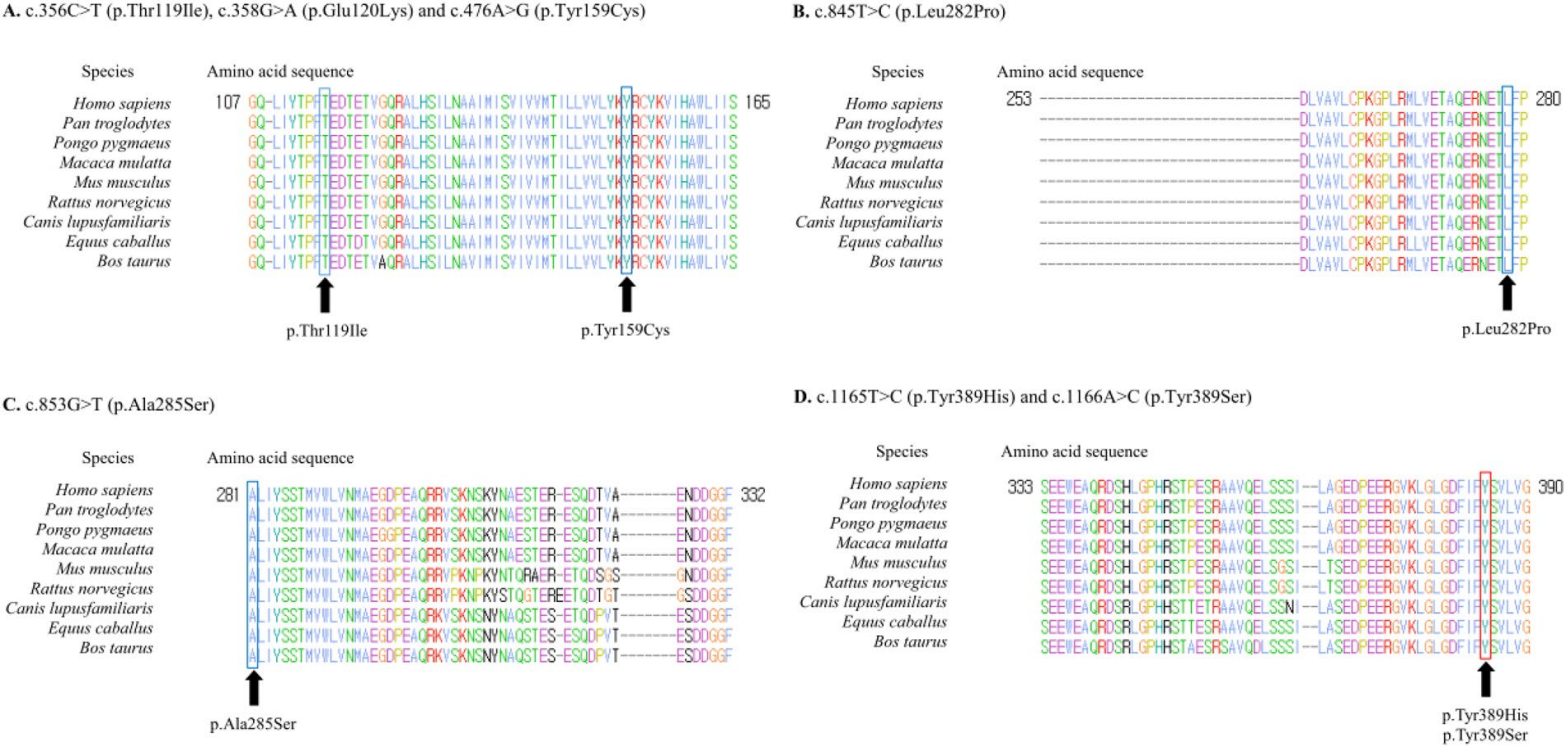
Evolutionary conservation of the amino acid residues for LPV (blue) and VUS (red) sites. Multiple sequence alignment shows the amino acid sites of four LPVs and two VUSs, (**A**) c.356 C > T (p.Thr119Ile), c.358 G > A (p.Glu120Lys) and c.476 A > G (p.Tyr159Cys), (**B**) c.845 T > C (p.Leu282Pro), (**C**) c.853 G > T (p.Ala285Ser), (**D**) c.1165 T > C (p.Tyr389His) and c.1166 A > C (p.Tyr389Ser).

A total of 6 of the 28 patients carried pathogenic or likely pathogenic variants in the *PSEN1* gene (21.4%, 6/28). Two pathogenic variants (PV), four likely pathogenic variants (LPV), and two variants of unknown significance (VUS) were identified. According to the age of onset, the *PSEN1* variants were found in four out of eight patients (50.0%) less than 50 years old at the age of onset and five out of 18 patients (27.8%) less than 60 years old at the age of onset. Two pathogenic variants (p.Glu120Lys and p.Ser170Phe) were detected in Patients 1 and 2 with onset ages of 34 and 28, respectively. Four LPVs, p.Thr119Ile, p.Tyr159Cys, p.Leu282Pro, and p.Ala285Ser, were found in Patients 3, 4, 5, and 6 with onset ages of 58, 59, 40, and 54, respectively. The two VUSs were p.Tyr389His, and p.Tyr389Ser which involved the same amino acid residue. None of the patients with *PSEN1* variants carried *APOE* ε4/ε4 alleles but one patient with LPV p.Tyr159Cys and two patients with VUSs had *APOE* ε3/ε4 alleles and one patient with PV p.Glu120Lys had *APOE* ε2/ε3 alleles. All VUSs were missense variants predicted to be damaging by PolyPhen-2 or SIFT and had GERP scores higher than 4 and CADD scores higher than 15, suggesting evolutionary conservation. The LPVs and the VUSs were not found in gnomAD (https://gnomad.broadinstitute.org/) or showed extremely low frequency in a non-neuro cohort (p. Tyr159Cys, 0.000005). In addition, all the LPVs and VUSs were not identified in 100 ethnically-matched normal control subjects, or 400 in-house controls. Two VUSs were located on the seventh transmembrane domain of the *PSEN1* gene (Table 1). Four LPVs and two VUSs occurred at amino acid residues which are highly conserved across species, including *Homo sapiens* and other mammals (Fig. 1).

**Table 1:** Identified variants of the *PSEN1* gene in 8 EOFAD patients.

Patient No.	Exon	Nucleotide change	Amino acid change	Protein domain	*APOE* genotype	*In silico* analysis		rs number	GERP score	CADD score	gnomAD^*^ (non-neuro cohort)	ALZFORUM Mutation Database^†^
						PolyPhen-2 (Probabilistic score)	SIFT (Tolerance index)					
**Pathogenic variants**												
1	5	c.358 G > A	p.Glu120Lys	NA	ε2/ε3	Probably damaging (1.000)	Not tolerable (0.00)	rs63750800	4.74	27.0	NA	Pathogenic
2	6	c.509 C > T	p.Ser170Phe	3^rd^ TM	ε3/ε3	Probably damaging (1.000)	Not tolerable (0.00)	rs63750577	4.57	25.5	0.000024	Pathogenic
**Likely pathogenic variants**												
3	5	c.356 C > T	p.Thr119Ile	NA	ε3/ε4	Probably damaging (0.973)	Tolerable (0.11)	NA	4.74	24.0	NA	Pathogenic
4	5	c.476 A > G	p.Tyr159Cys	NA	ε3/ε4	Probably damaging (1.000)	Not tolerable (0.00)	rs778630379	4.65	28.3	0.000005	NA
5	8	c.845 T > C	p.Leu282Pro	NA	ε3/ε3	Probably damaging (0.998)	Not tolerable (0.00)	NA	5.70	28.5	NA	NA
6	8	c.853 G > T	p.Ala285Ser	NA	ε3/ε3	Probably damaging (1.000)	Tolerable (0.14)	NA	5.82	25.9	NA	NA
**Variants of unknown significance**												
7	11	c.1165 T > C	p.Tyr389His	7^th^ TM	ε3/ε4	Probably damaging (0.998)	Not tolerable (0.00)	NA	4.54	26.9	NA	NA
8	11	c.1166 A > C	p.Tyr389Ser	7^th^ TM	ε3/ε3	Probably damaging (0.999)	Not tolerable (0.00)	NA	4.53	26.8	NA	NA

EOAD = Early onset Alzheimer’s disease; NA = not applicable; TM = transmembrane domain.

*gnomAD, http://gnomad.broadinstitute.org/.

^†^ALZFORUM Mutation Database, https://www.alzforum.org/mutations/psen-1.

### Clinical and neuroimaging findings

**Figure 2:**
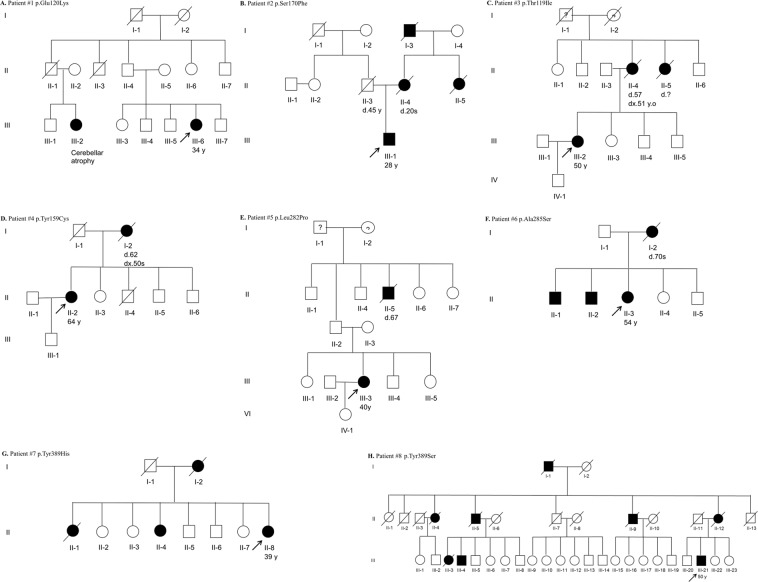
Pedigrees of EOFAD patients with *PSEN1* variants.

Table 2 provides a summary of clinical and neuroimaging findings for the eight patients with *PSEN1* variants. The mean age of onset was 44.4 years (range, 28 to 59) and all patients had a family history of dementia (Fig. 2). Patient 1 with p.Glu120Lys showed memory impairment, visuospatial dysfunction, language impairment such as anomia, psychiatric symptoms, cerebellar ataxia, and parkinsonism. Patient 2 with p.Ser170Phe also showed memory impairment, biparietal dysfunction (visuospatial dysfunction and acalculia), and myoclonus. Patient 3 with p.Thr119Ile and Patient 5 with p.Leu282Pro presented depression and apathy as well as memory impairment and visuospatial dysfunction. Patient 4 with p.Tyr159Cys showed aphasia, anxiety, and parkinsonism. Patient 6 with p.Ala285Ser and Patient 7 with p.Tyr389His presented with left parietal symptoms (apraxia or acalculia) and Patient 8 with p.Tyr389Ser showed aphasia and biparietal dysfunction.

**Table 2:** Clinical and neuroimaging findings in the 8 EOFAD patients with *PSEN1* gene variants.

Patient No.	Exon	Nucleotide change	Amino acid change	Sex	Onset age/ duration of illness (years)	Family history	Main clinical features	MMSE	MRI	FDG-PET	PiB-PET
1	5	c.358 G > A	p.Glu120Lys	F	34/5	+	memory impairment, visuospatial dysfunction, anomia, depression, apathy, abulia, delusion, cerebellar ataxia, parkinsonism, dystonia	16/30	mild diffuse cortical atrophy, left hippocampal atrophy	hypometabolism in the bilateral temporal lobes	PiB(+) 1.84
2	6	c.509 G > T	p.Ser170Phe	M	28/1	+	memory impairment, visuospatial dysfunction, anomia, acalculia, myoclonus	18/30	diffuse and biparietal lobe atrophy	hypometabolism in the bilateral parietal and temporal lobes	none
3	5	c.356 C > T	p.Thr119Ile	F	50/2	+	memory impairment, visuospatial dysfunction, depression, apathy	21/30	mild bilateral hippocampal atrophy	mild hypometabolism in the bilateral temporal lobes	none
4	5	c.476 A > G	p.Tyr159Cys	F	59/5	+	memory impairment, visuospatial dysfunction, aphasia, anxiety, parkinsonism	0/30	diffuse. biparietal and hippocampal atrophy	severe hypometabolism in bilateral fronto-parieto-temporal cortex	none
5	8	c.845 T > C	p.Leu282Pro	F	40/3	+	memory impairment, visuospatial dysfunction, abulia, apathy, depression	16/30	mild diffuse cortical atrophy	hypometabolism in the bilateral parietal and temporal cortex	none
6	8	c.853 G > T	p.Ala285Ser	F	54/5	+	memory impairment, acalculia, delusion, depression, anxiety	24/30	bilateral hippocampal atrophy	hypometabolism in the bilateral temporal cortex	PiB(+) 1.93
7	11	c.1165 T > C	p.Tyr389His	F	39/4	+	memory impairment, apraxia, acalculia, anomia, parkinsonism	26/30	mild diffuse cortical atrophy	hypometabolism in the bilateral parietal and temporal cortex	PiB(+)
8	11	c.1166 A > C	p.Tyr389Ser	M	50/3	+	memory impairment, visuospatial dysfunction, aphasia, acalculia, apraxia, simultagnosia, parkinsonism, apathy, abulia	7/30	mild diffuse cortical atrophy	hypometabolism in the bilateral parieto-temporal cortex	PiB(+)

EOAD = Early onset Alzheimer’s disease; NA = not applicable; MMSE = Mini-mental State Examination.

Brain MR images revealed diffuse cortical atrophy, including parietal lobe atrophy, and/or hippocampal atrophy. FDG-PET scans also displayed significant hypometabolism in the bilateral temporo-parietal regions. The four patients who had [^11^C] PiB-PET scans were all classified as PiB-positive (PiB+) using the measured global PiB uptake ratio values.

## Discussion

In this study, we investigated the frequency of *PSEN1* gene variants in 28 clinically suspicious EOFAD patients selected from 1,101 AD patients in Korea. We identified eight variants in the *PSEN1* gene, two PVs located at exon 5 (p.Glu120Lys) and exon 6 (p.Ser170Phe), four LPVs located at exon 5 (p.Thr119Ile, p.Tyr159Cys) and exon 8 (p.Leu282Pro p.Ala285Ser), and two VUSs located at exon 11 (p.Tyr389His, p.Tyr389Ser). Their phenotypes were consistent with those of previously reported cases and included early onset, frequent parietal symptoms, movement disorders, and rapid progression. Our findings provide insight to better understand the genetic background in Korean EOFAD patients.

The strength of our study was that clinically suspicious EOFAD patients were chosen from a consecutively recruited large sample of patients who had standardized phenotyping for AD and underwent genetic testing for *PSEN1*. Since *PSEN1* gene analysis was not possible for all patients because of ethical problems and cost-effectiveness, we believe that our selection strategy was practical. However, we acknowledge some limitations. First, we were unable to acquire DNA samples from other affected and unaffected family members either due to the absence of living affected relatives or the refusal for genetic analysis. Therefore, segregation analysis could not be performed. Second, we could not confirm AD pathology using brain autopsies. However, four cases among the eight *PSEN1* confirmed patients had [^11^C] PiB-PET scans and were classified as PiB+ which were compatible with AD.

In the present study, *PSEN1* gene variants were detected in eight AD patients. The frequency of *PSEN1* gene variants in AD patients was 0.7% (8/1101) and might be reasonable considering that patients who are autosomal dominant inherited are less than 1% of all AD patients^4,7^. EOFAD with onset from 30 to 60 years of age accounts for 1 to 6% of all AD cases^13^. Approximately 60% of EOAD cases had multiple first-to-second degree affected relatives and 13% were inherited in an autosomal dominant manner^14^. Among EOFAD patients, mutations in *PSEN1* genes have been reported in 55% (17/31) of the patients in the United Kingdom and 59% (20/39) in a French cohort^14,15^. However, the proportion of *PSEN1* mutations was lower in Chinese (15.2%, 4/32) and Polish cohorts (17%, 7/41)^16,17^. In Korea, 1.9% (2/104) of the EOAD patients carried a PV or LPV in the *PSEN1* gene^18^. The variable frequency of *PSEN1* pathogenic variants might be due to differences in subject inclusion criteria, such as the age of onset, the number of family members, and the degree of relatives with AD.

In this study, we identified two PVs, p.Glu120Lys in Patient 1 and p.Ser170Phe in Patient 2. These two are the first PVs identified in Korean patients with AD. The p.Glu120Lys variant was detected in a Danish AD patient with spastic paraparesis who was 43 at the age of onset^19^ and a Jewish-Israeli family with early-onset Alzheimer disease^20^. Missense variants altering the 120^th^ glutamic acid to lysine or asparagine have been reported in patients with early-onset or familial Alzheimer’s disease^19,21,22^. Our patient presented with memory impairment, anomia, depression, and apathy at 34 years old and also showed movement disorders, including cerebellar ataxia, parkinsonism, and dystonia. The p.Ser170Phe variant was originally described in an English family with EOFAD in which the onset of memory loss began at 26 to 27 years of age and the average duration of the disease was 11 years before death^23^. Since then, two more cases were reported in Polish and Italian patients^24,25^. These two cases showed very early-onset AD before the third decade of life. Consistent with previous cases, our patient with the p.Ser170Phe (Patient 2) variant developed symptoms at 28 years of age and showed a very rapid disease progression.

Among four LPVs, three LPVs have been reported as different amino acid changes in the same codons as those causing familial AD: the tyrosine at codon 159, the leucine at codon 282, and the alanine at codon 285. Previously a missense variant affecting the tyrosine at codon 159 (p.Tyr159Phe) was reported in a 43-year-old female with familial AD^26^. In the current study, Patient 4 with the p.Tyr159Cys variant showed parkinsonism, as well as cognitive impairment, with age of onset at 59. Different amino acid changes in the leucine at codon 282 in variants p.Leu282Phe, p.Leu282Arg, and p.Leu282Val have been repeatedly described as a cause of familial AD^27–29^. Similar clinical features were observed in patients with an amino acid alteration in the leucine at codon 282, who had symptom onset in their forties, late myoclonus, and seizures^30^. Patient 5 carried the p.Leu282Pro variant and was 40 years old at the age of onset, however, this patient did not show myoclonus or seizures until three years after the onset of AD. There are three prior reports of variants (p.Ala285Val) affecting the alanine at codon 285^31–33^. The mean age at onset was 46.7 and the patients had a relatively indolent course (range, 8–19 years) with progressive global cognitive impairment^32,33^. In the present study, Patient 6, carrying p.Ala285Ser, showed an insidious personality change, including delusion, depression, and anxiety, and progressive memory impairment, which was a similar clinical presentation as in the previous studies.

One LPV, the p.Thr119Ile variant in exon 5, was reported in EOAD patients^34,35^. Patient 3 who carried this variant showed an age at onset of 50 years. Our patient began with prominent memory impairment and depression, followed by visuospatial dysfunction and global cognitive impairment without atypical features. There has been no prior report of a sequence variant affecting this region.

We identified a VUS of C to T and A at codon 389 in the *PSEN1* gene which resulted in the substitution of tyrosine with histidine (p.Tyr389His) in Patient 7 and serine (p.Tyr389Ser) in Patient 8. Patient 7 had variant p.Tyr389His and showed memory impairment, apraxia, acalculia, and parkinsonism with an age of onset at 39 years. Patient 8, carrying p.Tyr389Ser, showed memory impairment, aphasia, apraxia, acalculia, parkinsonism, and apathy with an age of onset at 50 years.

To date, approximately 200 different variants discovered in the *PSEN1* gene have been associated with AD and the variants have revealed heterogeneity in clinical phenotypes^36^. EOFAD patients with PVs or LPVs in the *PSEN1* gene predominantly showed memory, visuospatial, and language dysfunctions. Along with cognitive impairments, atypical clinical features may be present in the course of the disease, such as behavioral and psychiatric features, parkinsonism, myoclonus, epileptic seizures, spastic paraparesis, aphasia, and cerebellar ataxia^5,36–38^. Clinical features of EOFAD seem to be different from those of sporadic AD, which are early onset, frequent parietal symptoms, movement disorders, and rapid progression.

In conclusion, we identified two PVs, four LPVs, and two VUSs in the *PSEN1* gene in eight EOFAD patients. Although variants in the *PSEN1* gene are a rare cause of AD, the identification of *PSEN1* PVs will contribute to a better understanding of the genetic background in Korean EOFAD patients. Furthermore, these findings could apply not only to identification of novel targets for genetic testing but to development of preventative and curative therapies for AD. Further clinical, biochemical, and molecular studies are needed to establish better genotype-phenotype correlations between EOFAD symptoms and *PSEN1* variants.

## Data Availability

The data that support the findings of this study are available on request from the corresponding author. The data are not publicly available due to privacy restrictions.

## References

[1] Ferri, C. *et al*. World Alzheimer Report 2014 Dementia and Risk Reduction An AnAlysis of pRotective AnD moDifiAble fActoRs EXECUTIVE SUMMARY dr Maëlenn Guerchet dr Matthew prina. (2014).

[2] Blennow K, de Leon MJ, Zetterberg H (2006). Alzheimer’s disease. Lancet (London, England).

[3] McMurtray AM (2006). Family history of dementia in early-onset versus very late-onset Alzheimer’s disease. Int. J. Geriatr. Psychiatry.

[4] Cruts M, Theuns J, Van Broeckhoven C (2012). Locus-specific mutation databases for neurodegenerative brain diseases. Hum. Mutat..

[5] Zou Z, Liu C, Che C, Huang H (2014). Clinical genetics of Alzheimer’s disease. Biomed Res. Int..

[6] Cruts M, Van Broeckhoven C (1998). Presenilin mutations in Alzheimer’s disease. Hum. Mutat..

[7] Nussbaum RL, Ellis CE (2003). Alzheimer’s disease and Parkinson’s disease. N. Engl. J. Med..

[8] Moulder KL (2013). Dominantly Inherited Alzheimer Network: facilitating research and clinical trials. Alzheimers. Res. Ther..

[9] McKhann G (1984). Clinical diagnosis of Alzheimer’s disease: report of the NINCDS-ADRDA Work Group under the auspices of Department of Health and Human Services Task Force on Alzheimer’s Disease. Neurology.

[10] McKhann GM (2011). The diagnosis of dementia due to Alzheimer’s disease: recommendations from the National Institute on Aging-Alzheimer’s Association workgroups on diagnostic guidelines for Alzheimer’s disease. Alzheimers. Dement..

[11] Ahn H-J (2010). Seoul Neuropsychological Screening Battery-dementia version (SNSB-D): a useful tool for assessing and monitoring cognitive impairments in dementia patients. J. Korean Med. Sci..

[12] Richards S (2015). Standards and guidelines for the interpretation of sequence variants: a joint consensus recommendation of the American College of Medical Genetics and Genomics and the Association for Molecular Pathology. Genet. Med..

[13] Bekris LM, Yu C-E, Bird TD, Tsuang DW (2010). Genetics of Alzheimer disease. J. Geriatr. Psychiatry Neurol..

[14] Campion D (1999). Early-onset autosomal dominant Alzheimer disease: prevalence, genetic heterogeneity, and mutation spectrum. Am. J. Hum. Genet..

[15] Janssen JC (2003). Early onset familial Alzheimer’s disease: Mutation frequency in 31 families. Neurology.

[16] Jiao B (2014). Mutational analysis in early-onset familial Alzheimer’s disease in Mainland China. Neurobiol. Aging.

[17] Zekanowski C (2003). Mutations in presenilin 1, presenilin 2 and amyloid precursor protein genes in patients with early-onset Alzheimer’s disease in Poland. Exp. Neurol..

[18] An SS (2016). A genetic screen of the mutations in the Korean patients with early-onset Alzheimer’s disease. Clin. Interv. Aging.

[19] Lindquist SG, Schwartz M, Batbayli M, Waldemar G, Nielsen JE (2009). Genetic testing in familial AD and FTD: mutation and phenotype spectrum in a Danish cohort. Clin. Genet..

[20] Reznik-Wolf H (1998). Germline mutational analysis of presenilin 1 and APP genes in Jewish-Israeli individuals with familial or early-onset Alzheimer disease using denaturing gradient gel electrophoresis (DGGE). Eur. J. Hum. Genet..

[21] Reznik-Wolf H (1996). A novel mutation of presenilin 1 in familial Alzheimer’s disease in Israel detected by denaturing gradient gel electrophoresis. Hum. Genet..

[22] Ryan NS (2016). Clinical phenotype and genetic associations in autosomal dominant familial Alzheimer’s disease: a case series. Lancet. Neurol..

[23] Snider BJ (2005). Novel presenilin 1 mutation (S170F) causing Alzheimer disease with Lewy bodies in the third decade of life. Arch. Neurol..

[24] Golan MP (2007). Early-onset Alzheimer’s disease with a de novo mutation in the presenilin 1 gene. Exp. Neurol..

[25] Piccini A (2007). Association of a presenilin 1 S170F mutation with a novel Alzheimer disease molecular phenotype. Arch. Neurol..

[26] Kerchner GA, Holbrook K (2012). Novel presenilin-1 Y159F sequence variant associated with early-onset Alzheimer’s disease. Neurosci. Lett..

[27] Aldudo J, Bullido MJ, Arbizu T, Oliva R, Valdivieso F (1998). Identification of a novel mutation (Leu282Arg) of the human presenilin 1 gene in Alzheimer’s disease. Neurosci. Lett..

[28] Dermaut B (2001). Cerebral amyloid angiopathy is a pathogenic lesion in Alzheimer’s disease due to a novel presenilin 1 mutation. Brain.

[29] Hamaguchi T, Morinaga A, Tsukie T, Kuwano R, Yamada M (2009). A novel presenilin 1 mutation (L282F) in familial Alzheimer’s disease. Journal of neurology.

[30] Gomez-Tortosa E (2010). Clinical-genetic correlations in familial Alzheimer’s disease caused by presenilin 1 mutations. J. Alzheimers. Dis..

[31] Rogaev EI (1995). Familial Alzheimer’s disease in kindreds with missense mutations in a gene on chromosome 1 related to the Alzheimer’s disease type 3 gene. Nature.

[32] Aoki M (1997). A presenilin-1 mutation in a Japanese family with Alzheimer’s disease and distinctive abnormalities on cranial MRI. Neurology.

[33] Ikeda M (1996). The clinical phenotype of two missense mutations in the presenilin I gene in Japanese patients. Ann. Neurol..

[34] Giau VV (2019). Genetic analyses of early-onset Alzheimer’s disease using next generation sequencing. Sci. Rep..

[35] Itzcovich Tatiana, Chrem-Méndez Patricio, Vázquez Silvia, Barbieri-Kennedy Micaela, Niikado Matías, Martinetto Horacio, Allegri Ricardo, Sevlever Gustavo, Surace Ezequiel I. (2020). A novel mutation in PSEN1 (p.T119I) in an Argentine family with early- and late-onset Alzheimer's disease. Neurobiology of Aging.

[36] Larner AJ (2013). Presenilin-1 mutations in Alzheimer’s disease: an update on genotype-phenotype relationships. J. Alzheimers. Dis..

[37] Filley CM (2007). The genetics of very early onset Alzheimer disease. Cogn. Behav. Neurol..

[38] Larner AJ, Doran M (2006). Clinical phenotypic heterogeneity of Alzheimer’s disease associated with mutations of the presenilin-1 gene. J. Neurol..

